# Probiotic *Aspergillus oryzae* produces anti-tumor mediator and exerts anti-tumor effects in pancreatic cancer through the p38 MAPK signaling pathway

**DOI:** 10.1038/s41598-021-90707-4

**Published:** 2021-05-26

**Authors:** Hiroaki Konishi, Shotaro Isozaki, Shin Kashima, Kentaro Moriichi, Satoshi Ichikawa, Kazuki Yamamoto, Chikage Yamamura, Katsuyoshi Ando, Nobuhiro Ueno, Hiroaki Akutsu, Naoki Ogawa, Mikihiro Fujiya

**Affiliations:** 1 Department of Gastroenterology and Advanced Medical Sciences, Asahikawa Medical University, 2-1-1-1, Midorigaoka, Asahikawa, Hokkaido, 078-8510 Japan; 2 Division of Metabolism and Biosystemic Science, Gastroenterology, and Hematology/Oncology, Department of Medicine, Asahikawa Medical University, Hokkaido, Japan; 3 Faculty of Pharmaceutical Science, Hokkaido University, Hokkaido, Japan; 4 Global Station for Biosurfaces and Drug Discovery, Global Institution for Collaborative Research and Education (GI-CoRE), Hokkaido University, Hokkaido, Japan; 5 Center for Advanced Research and Education, Asahikawa Medical University, Hokkaido, Japan

**Keywords:** Cancer, Microbiology

## Abstract

Intake of probiotics or fermented food produced by some probiotic bacteria is believed to exert anti-tumor functions in various cancers, including pancreatic cancer, because several studies have demonstrated the anti-tumor effects of probiotic bacteria in vitro and in vivo in animal carcinogenesis models. However, the mechanisms underlying the anticancer effects of probiotics on pancreatic cancer have not been clarified. In this study, we assessed the anti-tumor effects of probiotic bacteria against pancreatic cancer cells. Among the known probiotic bacteria, *Aspergillus oryzae* exhibited a strong pancreatic tumor suppression effect. The culture supernatant of *A. oryzae* was separated by HPLC. Heptelidic acid was identified as an anti-tumor molecule derived from *A. oryzae* by LC–MS and NMR analysis. The anti-tumor effect of heptelidic acid was exhibited in vitro and in vivo in a xenograft model of pancreatic cancer cells. The anti-tumor effect of heptelidic acid was exerted by the p38 MAPK signaling pathway. Heptelidic acid traverses the intestinal mucosa and exerts anti-tumor effects on pancreatic cancer cells. This is a novel anti-tumor mechanism induced by beneficial bacteria against pancreatic cancer in which bacterial molecules pass through the intestinal tract, reach the extra-intestinal organs, and then induce apoptosis via an inducible signaling pathway.

## Introduction

Probiotics are live microorganisms that confer a health benefit, including anti-tumor effects, on the consumer when they are administered in adequate amounts^1^. It is strongly believed that fermented foods produced by probiotic bacteria, including yoghurt and soybean paste, exert anti-tumor effects through the modification of the consumer’s internal condition, such as their immunity and nutritional status^2–5^.

Over the past 10 years, several probiotic-derived anti-tumor molecules have been identified from cultures of probiotics. *Lactobacillus casei* ATCC 334 releases the antimicrobial peptides (AMPs), m2163 and m2386, which are toxic to certain microorganisms. These peptides induce apoptosis of colorectal cancer cells through the induction of Fas-mediated death signaling^6^. *L. brevis* SBL88-derived inorganic polyphosphate, which bacteria utilize as an energy source for ATP^7,8^, activates extracellular-signal-regulated kinase (ERK) signaling, resulting in apoptosis of colorectal cancer cells^9^. We also identified ferrichrome as tumor-suppressive molecule derived from culture supernatant of *L.casei* ATCC334^10^. Ferrichrome exhibited the anti-tumor effect through inducing apoptosis of colorectal cancer cells and gastric cancer cells by the activation of c-Jun N-terminal kinase (JNK)-mediated DDIT3 signaling^11,12^. Recently, we showed that ferrichrome exerts anti-tumor effects mediating p53 activation in pancreatic cancer cells^13^. These investigations suggest that probiotics fight cancer cells by producing anti-tumor agents as well as immunopotentiating.

Pancreatic cancer is recognized as the “king” of cancer, with a 5-year survival rate under 10%^14^. Chronic pancreatitis and diabetes as well as genetic mutations in KRAS and TP53 are thought to be associated with the pathogenesis of pancreatic cancer, although the precise causes of pancreatic cancer remain unclear^15,16^. Novel therapeutic approaches for pancreatic cancer, including unfolded protein response (UPR) inhibitor^17^ and multi-targeted tyrosine kinase inhibitor^18^, have been developing over the past decade. Recently, it was indicated that long non-coding RNA is also associated with pathogenesis and drug resistance of pancreatic cancer^19^. These findings suggest that the factors associated with the pathogenesis of pancreatic cancer as well as therapeutic approaches, including its treatment and prevention, need to be clarified in order to fully understand the pancreatic cancer-associated factors.

Previous investigations indicated that disruption of the microflora of the gastrointestinal tract, including the colon and duodenum, frequently occurs in pancreatic cancer patients, and some treatments, including probiotics and fecal microbiota transplantation, are expected to be useful for pancreatic cancer treatment^20,21^. Importantly, the bacterial body cannot traverse the gastrointestinal tract. Thus, bacteria cannot directly attach to extra-intestinal organs, including the pancreas. However, some groups have reported that the oral intake of probiotics and fermented food produced by probiotic bacteria, including *Lactobacillus* and *Aspergillus*, resulted in an anti-tumor effect and increased the anti-tumor effects of chemotherapy in animal carcinogenesis models of pancreatic cancer^22,23^. Thus, some species of bacteria produce anti-tumor molecules against pancreatic cancer cells in the intestinal tract, which then pass out of the intestinal track to other organs, resulting in anti-tumor effects against pancreatic cancer.

As described above, we found that probiotic-derived ferrichrome inhibited pancreatic cancer cell progression. When ferrichrome was intraperitoneally administered to a xenograft model of pancreatic cancer cells, strong tumor-suppressive functions were observed, suggesting that intraperitoneally administered ferrichrome was transmitted through mammalian vascular endothelial cells, entered the blood stream and reached the pancreatic cancer cells. This supports the notion that the anti-tumor effects of fermented food of probiotic bacteria against pancreatic cancer are induced by some sort of bacteria-derived molecules that passes through the gastrointestinal tract. However, anti-tumor mediators against pancreatic cancer produced by probiotic bacteria and their anti-tumor mechanisms remain unclear.

We explored the mechanism underlying the inhibition of pancreatic cancer progression by probiotic products.

## Materials and methods

### Cell culture

The human pancreatic cancer cell lines SUIT2 (Health Science Research Resources Bank), Panc-1 (American Tissue Culture Collection [ATCC]) and MIA-PaCa-II (JCRB Cell Bank) were grown in high-glucose Dulbecco’s Modified Eagle’s Medium (DMEM) (SUIT2 and MIA-PaCa-II) or Roswell Park Memorial Institute (RPMI) 1640 (PANC-1) supplemented with 10% (vol/vol) fetal bovine serum (FBS), 2 mM l-glutamine, 50 U/ml penicillin and 50 µg/ml streptomycin in a humidified atmosphere containing 5% CO_2_. The cells were plated at a density of 10^5^ cells/cm^2^.

### Microorganisms

*A. oryzae* ATCC42149, *S. cereviceae* ATCC9763*, L. casei* ATCC334*, L. acidophillus* ATCC314*, L. fermentum* ATCC23271*, L. rhamnosus GG* (LGG) ATCC53103*, B. infantis* ATCC15697*, B. lactis* ATCC700541*, B. longum* ATCC15707 *and B. adlescentis* ATCC15703 were purchased from ATCC. Fungi (*A. oryzae*, *S. cereviceae*), Lactobacilli (*L. casei, L. acidophillus, L. fermentum,* LGG) and Bifidobacterium (*B. infantis, B. lactis, B. longum, B. adlescentis*) were cultured in YM broth (Difco Laboratories, Detroit, MN, USA), Man–Rogosa–Sharpe (MRS) broth (Difco Laboratories) and Clostoridial broth (Difco Laboratories), respectively, for 2–3 day at 37 °C. Each of the Lactobacilli was then cultured in DMEM for another day.

### Isolation of the tumor-suppressive molecule

The culture medium was centrifuged at 5000×*g* for 10 min to obtain the culture supernatant, which was then filtered through a 0.2-μm membrane. The culture supernatants were separated with a molecular weight cut-off (MWCO) spin column (GE Healthcare). The culture supernatant was separated using an AKTA Design HPLC system (GE Healthcare) with a Superdex peptide column (GE Healthcare) and eluted with distilled water at a flow rate of 1 mL/min. The fraction was applied to a Wakopak® Wakosil® C18 and C8 column (Wako Pure Chemical, Osaka, Japan) and eluted with 0.1% formic acid and 0.1% formic acid/acetonitrile in a linear gradient at a flow rate of 2.5 mL/min. The eluent was monitored by ultraviolet spectrophotometry at 210 nm.

### The SRB assay

The significance of cell density by samples was determined by the SRB assay. At 24 h before stimulation, the cells were seeded on 96-well microplates at 1.0 × 10^4^ cells/100 μL/well. Each sample was diluted with cell culture media and applied to the pancreatic cancer cells. At 24, 48 or 72 h after stimulation, the cells were fixed in 5% trichloroacetic acid (TCA) for 1 h at 4 °C and washed 4 times in distilled water. The microplates were then dehydrated at room temperature, stained in 100 μL/well of 0.057% (wt/vol) SRB powder/distilled water, washed 4 times in 0.1% acetic acid and re-dehydrated at room temperature. The stained cells were lysed in 10 mM Tris-buffer, and the optical density (OD) was measured at 510 nm.

### Mass spectrometry (MS)

A low-resolution liquid chromatography with tandem mass spectrometry (LC–MS) analysis was performed using a Prominence-I LC-2030C Plus, LC–MS-8040 (Shimadzu Corporation, Kyoto, Japan) and J’sphere ODS-M80 (150 × 4.6 mml., S-4 μm, 8 nm) as a C18 column. Elusion used an acetonitrile–water gradient (5–90%; 0.4 mL/min) at 30 °C and was monitored using an ultraviolet (UV) light detector (254 nm). High-resolution MS was performed using a Waters Xevo G2 QTof. Samples were eluted by acetonitrile.

### A nuclear magnetic resonance (NMR) analysis

The ^1^H NMR spectrum of the isolated compound was measured in CDCl_3_ solution and reported in parts per million (δ) relative to tetramethylsilane (0.00 ppm) as the internal standard using a JEOL JMM-ECS-400 spectrometer at room temperature.

### Compounds

Heptelidic acid and 5-FU were purchased from Adipogen Life Sciences, CA, USA, and Sigma-Aldrich, MO, USA, respectively.

### Western blotting

Total proteins were extracted from samples using a NP-40 Cell Lysis Buffer (Thermo Fisher Scientific, MA, USA) containing Complete protease inhibitor (Sigma Aldrich) and Halt Phosphatase Inhibitor Cocktail (Thermo Fisher Scientific). Equal amounts of protein were resolved using sodium dodecyl sulfate–polyacrylamide gel electrophoresis (SDS-PAGE) (12.5%), blotted onto a nitrocellulose membrane and then blocked in phosphate-buffered saline (PBS) with 0.05% (vol/vol) Tween 20 (T-PBS) containing 1% (wt/vol) bovine serum albumin (BSA). The blots were incubated overnight at 4 °C with primary antibodies. The primary antibodies of phospho-Akt (S473; #4051, T308; #4056), JNK (#9251), ERK (#4377), p38MAPK (#9215), GSK3β (#9336), cleaved caspase-3 (#9661) and PARP (#9452) were purchased from Cell Signaling Technology. All antibodies were diluted in 1/1000 in SuperBlock™ (TBS) Blocking Buffer (Thermo Fisher Scientific) and incubated with blots overnight at 4 °C. The blots were washed in TBS containing 0.05% Tween-20, incubated with HRP-conjugated secondary antibodies (R&D Systems, Minneapolis, MN, USA), washed in T-PBS and then developed using the Super-Signal West Pico enhanced chemiluminescence system (Thermo Fisher Scientific). The average protein expression was normalized to the actin expression (BD Transduction Laboratories, Lexington, KY, USA).

### Animal experiments

All experimental methods were carried out in accordance with relevant guidelines and regulations. The protocols of the animal experiments were approved by the Asahikawa Medical University Institutional Animal Care and Use Committee (Approved No; 20057). The described studies were carried out in compliance with the ARRIVE guidelines. BALB/c nude mice and BALB/c mice (male; age, 6–10 weeks; weight, 20–25 g) housed at 20–25 °C with 30–60% humidity under a 12:12-h light/dark cycle with ad libitum access to food and water were used for the xenograft experiment and intestinal loop study, respectively. Animal health and behavior were monitored on the drug treatment day. For sacrifice, 4–5% isoflurane was administered via inhalation to mice, and then cervical dislocation was performed under anesthesia. The death of mice was confirmed by monitoring respiratory and cardiac arrest.

### Xenografts

Male BALB/c nude mice were purchased from Charles River Laboratories Japan, Inc. SUIT-2 cells (2 × 10^6^ cells/35 μL PBS/tumor) were mixed with matrigel (15 μL/tumor), and 50 μL of cell suspension was injected subcutaneously into the back of BALB/c nude mice (6 weeks old). PBS or heptelidic acid (1 μg) was locally injected daily, starting the day after the injection of SUIT-2 cells. The tumor size was calculated by the following formula: Tumor size (mm^2^) = (major diameter) × (minor diameter).

The animal studies were approved by the Asahikawa Medical University Institutional Animal Care and Use Committee (Approved No; 20,057), and all procedures were performed in accordance with the relevant guidelines and regulations.

### Intestinal loop study

Male BALB/c mice were purchased from Charles River Laboratories Japan, Inc. BALB/c mice were sacrificed, and the small intestine was divided into 3 pieces, with each end ligated with silk sutures and the loops filled with DMEM supplemented with 10% (vol/vol) FBS, 2 mM l-glutamine, 50 U/ml penicillin and 50 µg/mL streptomycin containing heptelidic acid or 5-FU. Loops were incubated for 2, 6 and 18 h at 37 °C in a 5% CO_2_ incubator. The outside media were collected and filtrated by a 0.45-nm membrane filter. Collected media were administered to SUIT-2 cells, and the growth changes were monitored by an SRB assay.

### Terminal deoxynucleotidyl transferase dUTP nick-end labeling (TUNEL) staining

At 24 h before stimulation, SUIT-2 cells were plated on a Lab-Tek® Chamber Slide™ (ThermoFisher Scientific). Heptelidic acid was diluted by culture media to 1 μg/mL and applied to the cells. At 72 h after stimulation, the slides were fixed in 4% paraformaldehyde and washed extensively with PBS. The slides were stained using an In Situ Cell Death Detection Kit and TMR red (Roche Diagnostic, Indianapolis, IN, USA) according to the manufacturer’s instructions. The cells were mounted with an anti-fade mounting medium, and the TUNEL-positive cells were visualized by fluorescence microscopy (KEYENCE Corporation, Osaka, Japan).

### Statistical analyses

The assay data were analyzed using Student’s *t*-test. P values of < 0.05 were considered to indicate statistical significance.

## Results

### The tumor-suppressive effects of probiotic culture supernatant

**Figure 1:**
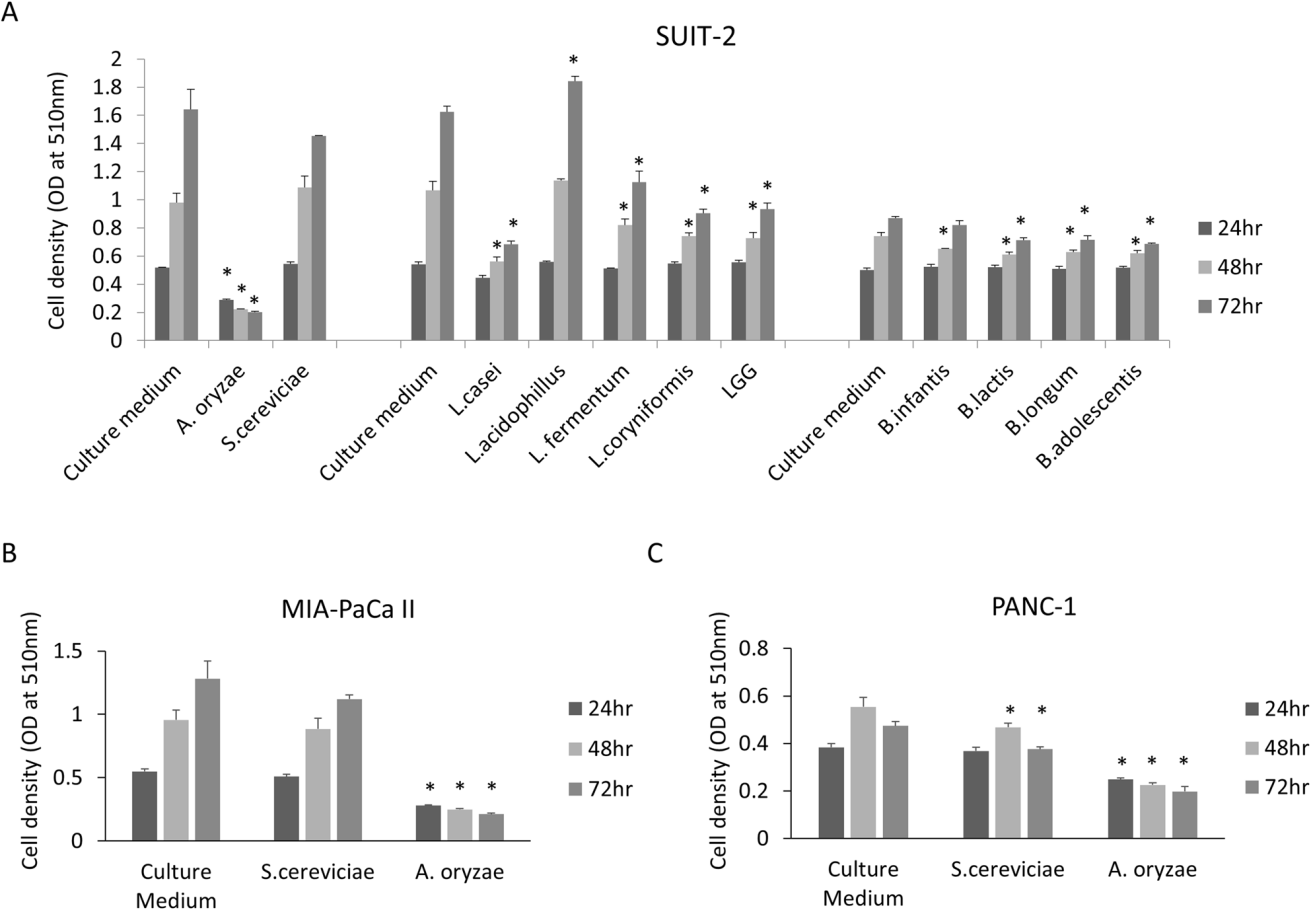
The culture supernatant of probiotic bacteria suppressed the pancreatic cancer cell growth. The bacteria-conditioned media was filtrated through a 0.22-μm-pore filter to eliminate the bacterial body and then diluted to 1/5 with cell culture medium. The SRB assay showed that the cell density of pancreatic cancer cells SUIT-2 (**A**), MIA-PaCa-II (**B**) and PANC-1 (**C**) was reduced by treatment with diluted conditioned media of probiotic bacteria, especially *A. oryzae*. The error bars show the standard deviation (S.D.) (n = 3).

To identify probiotics that exert anti-tumor effects in pancreatic cancer cells, 11 species of probiotics (2 fungi [*A. oryzae*, *S. cereviceae*], 5 Lactobacillus [*L. casei, L. acidophillus, L. fermentum, L. coryniformis*, LGG], 4 Biffidobacterium [*B. infantis, B. lactis, B. longun. B. adolescentis*]) were cultured in each media, and the culture supernatant was collected by centrifugation and membrane filtration. An SRB assay revealed a growth inhibition effect in SUIT-2 cells following treatment with the culture supernatant of *A. oryzae*, *L. casei, L. fermentum, L. coryniformis* or LGG (Fig. 1A). The culture supernatant of *A. oryzae*, which exhibited the strongest tumor-suppressive effect, was administered to other pancreatic cancer cell lines (MIA-PaCa-II and PANC-1 cells), and a strong tumor-suppressive effect was confirmed in these cells (Fig. 1B,C), suggesting that *A. oryzae* produced anti-tumor molecules against pancreatic cancer cells. These data suggest that *A. oryzae*-derived molecules inhibited the pancreatic cancer progression.

### The fractionation of culture supernatant of A. oryzae

**Figure 2:**
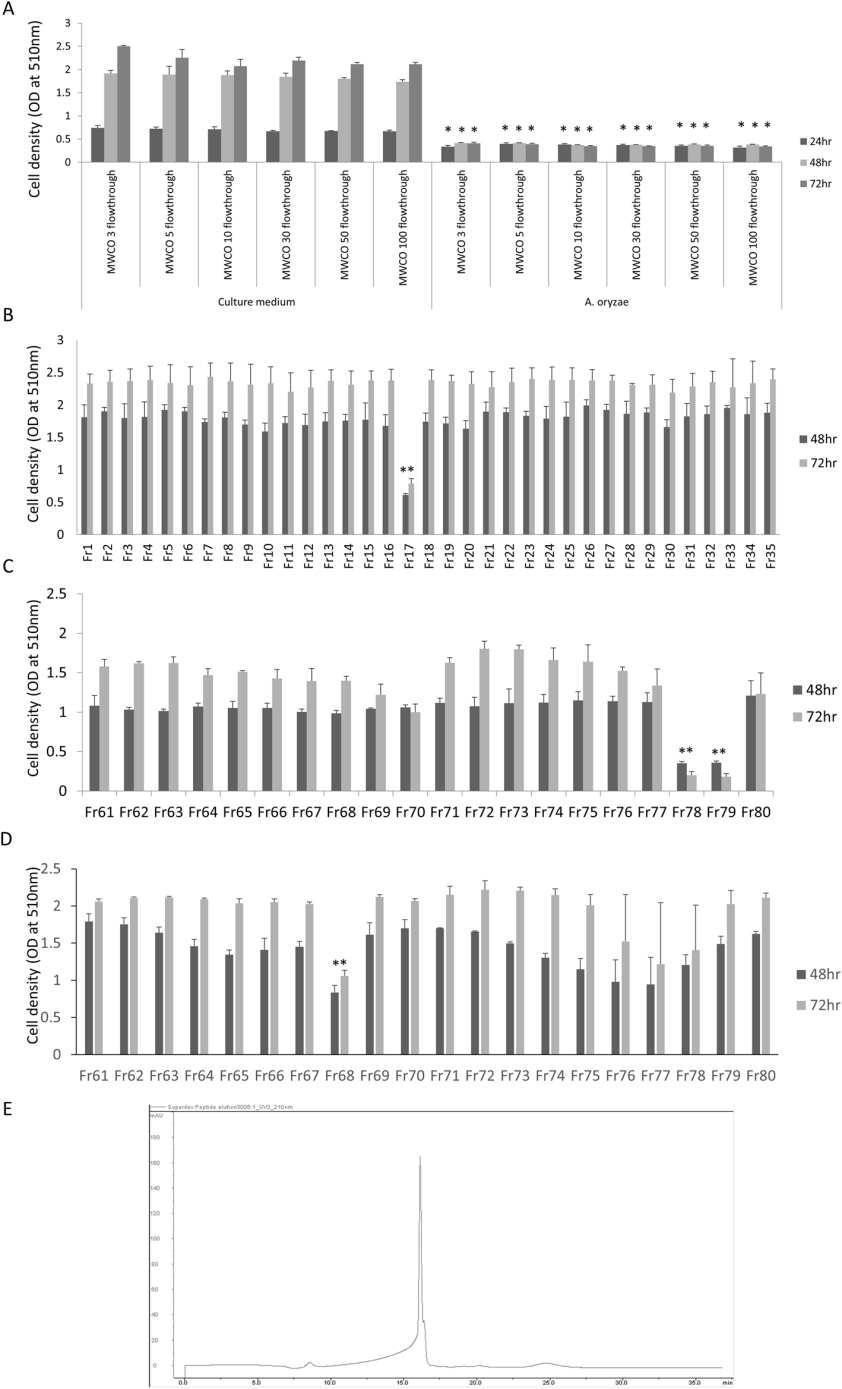
Fractionation of the culture supernatant of *A. oryzae*. An SRB assay showed that the culture supernatant of *A. oryzae*, which was separated by a 3-, 5-, 10-, 30- and 50-kDa molecular weight cut-off membrane filter, exerted a growth-suppression effect against SUIT-2 cells (**A**). The culture supernatant of *A. oryzae* was fractionated by size exclusion chromatography using a gel filtration column, and the tumor-suppressive function was detected in Fr 17th (**B**). The Fr 17th was separated by reverse-phase chromatography using a C18 column, and the tumor-suppressive effect was detected in Fr 78th and 79th (**C**). The mixture of Fr 78th and 79th was further separated by the C8 column, and a tumor-suppressive effect was observed in Fr 68th (**D**). The spectrum analysis using a gel filtration column showed a single peak of Fr 68th (**E**). The error bars show the standard deviation (S.D.) (n = 3).

To determine the size of the anti-tumor molecules derived from *A. oryzae*, the culture supernatant was separated by the MWCO membrane column. An SRB assay showed that the flowthrough fraction of 3 kDa maintained a tumor-suppressive effect against SUIT-2 cells (Fig. 2A), suggesting that the size of the anti-tumor molecule derived from *A. oryzae* was < 3 kDa.

To separate the low-molecular-weight fraction, a gel filtration column for the separation of low-molecular-weight molecules, such as peptide, was used for fractionation by high-performance liquid chromatography (HPLC). Fraction 17 (Fr17) showed the strong tumor-suppressive effect (Fig. 2B). To fractionate the collected fraction, reverse-phase chromatography using a C18 column was used. Surprisingly, the tumor-suppressive molecules derived from *A. oryzae* were strongly captured by the C18 column (Fr78, 79) (Fig. 2C). For the further separation of the collected fraction, a C8 column was used, and Fr68 showed a tumor-suppressive effect (Fig. 2D). Finally, the HPLC spectrum of the collected fraction was assessed, showing a single peak. This suggested that the anti-tumor molecules derived from *A. oryzae* were enriched in the collected fraction (Fig. 2E).

### Heptelidic acid was identified as a tumor-suppressive molecule of A. oryzae

**Figure 3:**
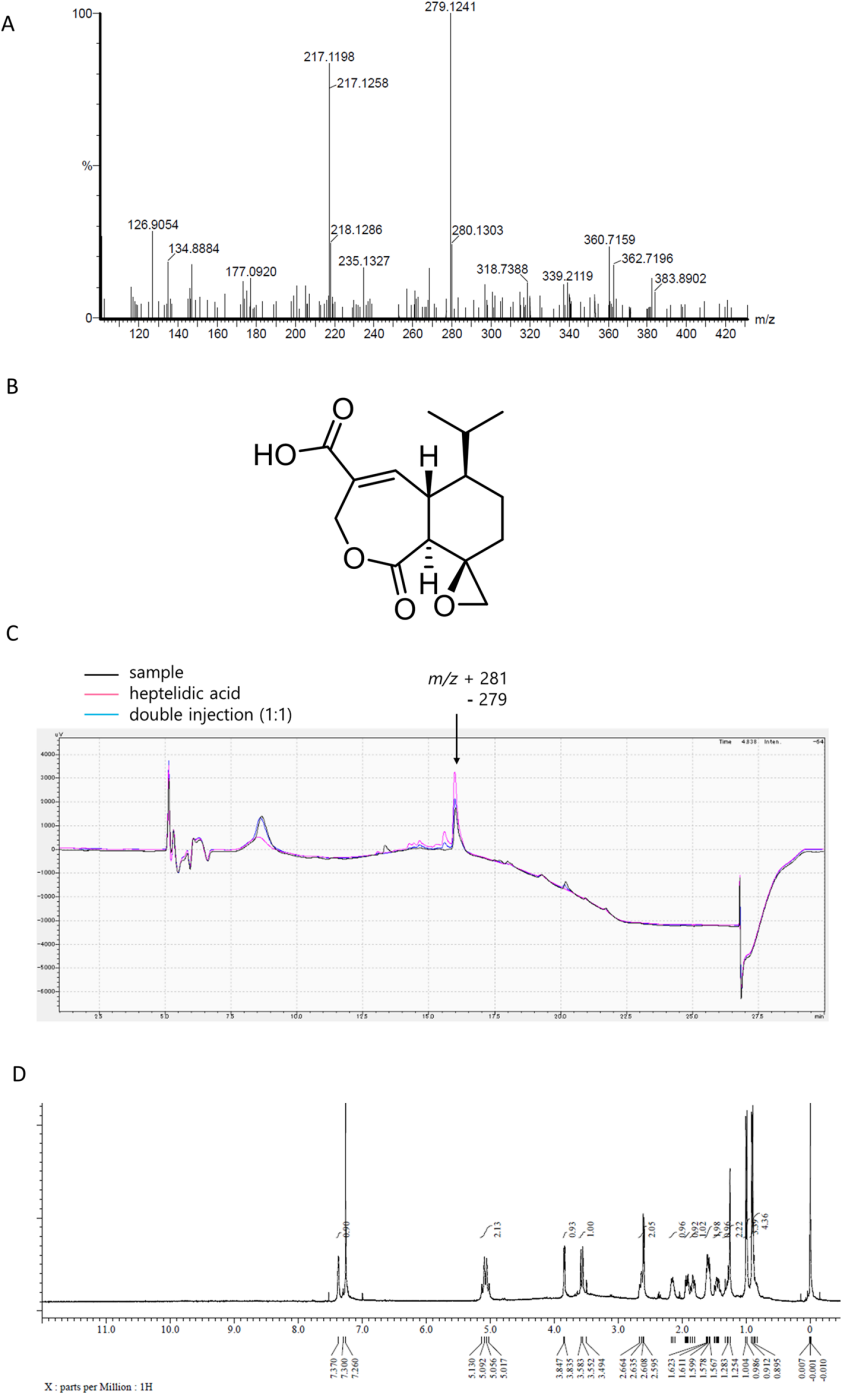
Heptelidic acid was contained in the separated fraction of the culture supernatant of *A. oryzae*. An LC-HRMS analysis showed the exact mass of the molecule contained in the final fraction (**A**). The chemical structure of heptelidic acid (**B**). The spectrum of LC confirmed heptelidic acid and the molecule contained in the final fraction to have the same retention time (**C**). The NMR analysis showed the spectrum of the molecule contained in the final fraction (**D**).

To clarify the tumor-suppressive molecules derived from *A. oryzae*, an LC- mass spectrometry (MS) analysis was performed. The spectrum of [M-H]^+^ = 281 was mainly detected (Supplementary Fig. S1). LC-high-resolution mass spectrometry (HRMS) analysis was performed to determine the exact mass of the tumor-suppressive molecules (Fig. 3A), and 11 formulae were highlighted as candidates (Table 1). These 11 formulae were searched in Pubchem, and heptelidic acid was found (https://pubchem.ncbi.nlm.nih.gov/compound/Heptelidic-acid; structure of heptelidic acid described in Fig. 3B). A previous investigation suggested that heptelidic acid was a metabolite of *A. oryzae*^24^. To determine whether or not heptelidic acid was contained in the fraction, LC and an NMR analysis were performed. LC analysis showed that the spectrum of the sample fraction was consistent with that of heptelidic acid (Fig. 3C). The NMR analysis showed that the spectrum of heptelidic acid^25^ corresponded with that of the sample fraction (Fig. 3D). These data suggested that heptelidic acid was the molecule derived from *A. oryzae* responsible for the inhibition of pancreatic cancer cell growth.

**Table 1:** The list of estimated anti-tumor molecules derived from *A. oryzae.*

Found Mass	Calc.Mass	PPM	Fomula	[M]+H	Estimated Mass
279.1241	279.1241	0	C16 H23 S2	280.1319	280.488
	279.1239	0.7	C8 H19 N6 O3 S	280.1218	280.347
	279.1246	− 1.8	C16 H15 N4 O	280.1324	280.331
	279.1236	1.8	C10 H16 N8 P	280.1314	280.2758
	279.1232	3.2	C15 H19 O5	280.1311	280.32
	279.1252	− 3.9	C8 H21 N6 O P2	280.133	280.2525
	279.1222	6.8	C9 H20 N4 O4 P	280.13	280.2648
	279.1262	− 7.5	C14 H20 N2 O2 P	280.1321	280.3078
	279.1266	− 9	C12 H23 O5 S	280.1344	280.379
	279.1214	9.7	C11 H25 N2 P2 S	280.1292	280.3505
	279.1269	− 10	C7 H20 N8 P S	280.1347	280.3348

### Heptelidic acid exhibited a tumor-suppressive effect against pancreatic cancer in vitro and in vivo

**Figure 4:**
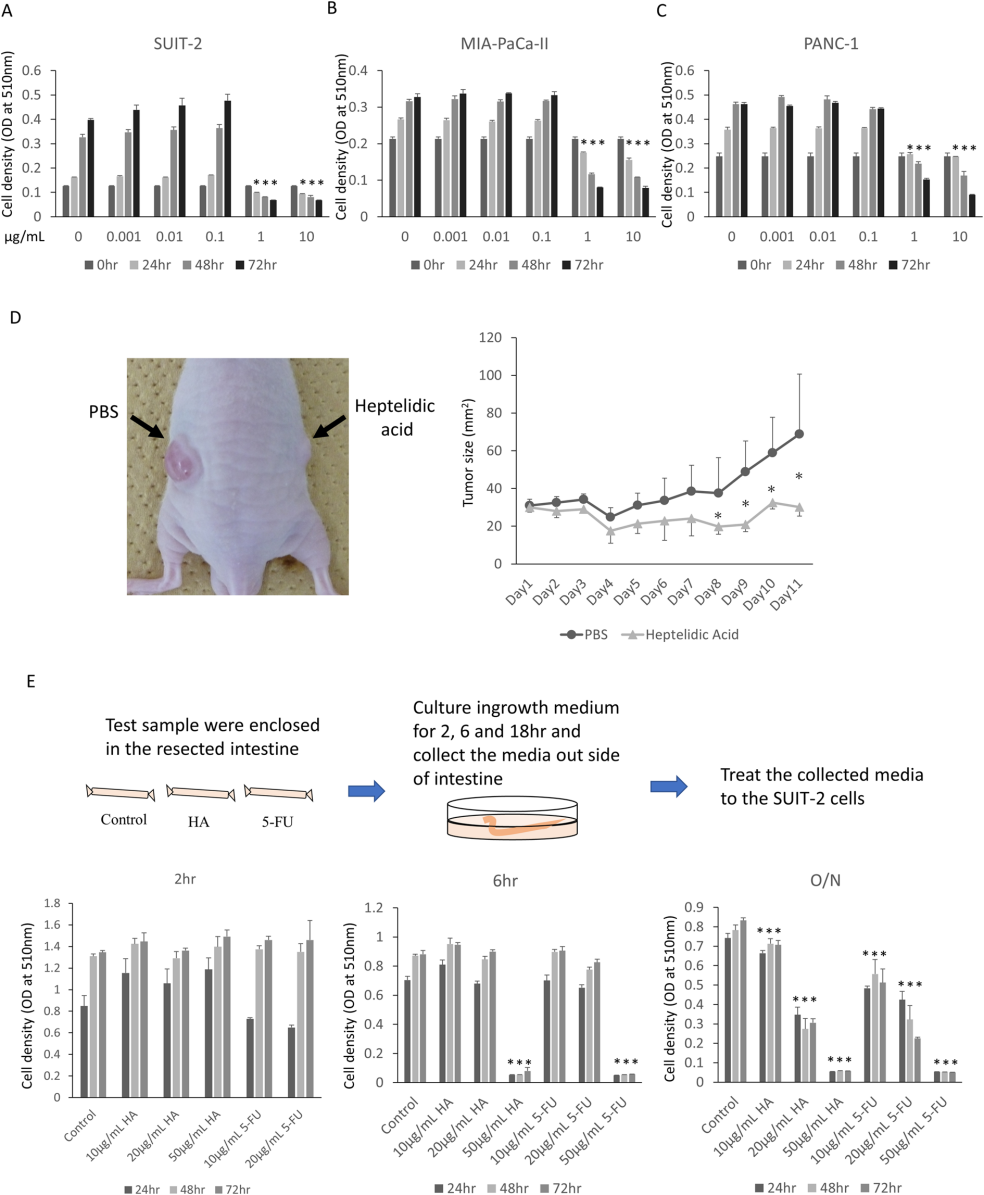
Heptelidic acid showed the anti-tumor effect in vitro and in vivo. The SRB assay showed that heptelidic acid exerted a growth-suppression effect against SUIT-2 (**A**), MIA-PaCa-II (**B**) and PANC-1 (**C**) cells in a concentration-dependent manner. SUIT-2 cells were transplanted into nude mice, and 10 μg of heptelidic acid was injected daily to the tumor. The growth of the tumor was significantly inhibited by treatment with heptelidic acid (**D**). Heptelidic acid or 5-FU was injected into the small intestine of mice and incubated in culture media for 2, 6 and 18 h. The SRB assay showed the anti-tumor effect of the media collected from outside the intestine enclosed with heptelidic acid or 5-FU (**E**). The error bars show the standard deviation (S.D.) (**A**,**B**,**C**,**E**; n = 3, **D**; n = 5).

To confirm the tumor-suppressive effects of heptelidic acid in pancreatic cancer cells, an SRB assay was performed. The growth of SUIT-2, MIA-PaCa-II and PANC-1 was significantly suppressed by heptelidic acid in a concentration-dependent manner (Fig. 4A–C). To confirm whether or not heptelidic acid exerts tumor-suppressive effects in vivo, SUIT-2 cells was transplanted into nude mice, and heptelidic acid was directly injected into the transplanted tumor daily. The tumor size was significantly suppressed by the administration of heptelidic acid (Fig. 4D). These data suggest that heptelidic acid exerts a tumor-suppressive effect in vitro and in vivo.

To clarify whether or not heptelidic acid is absorbed from the intestinal tract to affect the pancreas, the small intestine of mice was resected, and heptelidic acid or 5-fluorouracil (5-FU), which are used as oral anti-tumor agents in the clinical therapy for pancreatic cancer, was enclosed in the intestinal loop. The loops were incubated in the culture media and the outside media were administered to SUIT-2 cells, and then the growth changes were investigated. The SRB assay showed the growth-suppressing effect of the medium outside of heptelidic acid or the 5-FU enclosed intestinal loop in a concentration- and treatment time-dependent manner (Fig. 4E). This suggests that heptelidic acid inhibits the tumor progression in extra-intestinal organs, including the pancreas, via the intestinal absorption of the *A. oryzae*-derived tumor-suppressing molecule heptelidic acid and subsequent delivery of that molecule to extra-intestinal organs.

### Heptelidic acid exerted a tumor-suppressive effect through activating p38 MAPK signal transduction, thereby mediating apoptosis of the pancreatic cancer cells

To assess the mechanisms underlying the tumor-suppressive effect of heptelidic acid, the expression of the apoptosis-related molecule poly[ADP]-ribosepolymerase (PARP) and cell cycle-related molecules cyclin D1 and B1 was evaluated.

**Figure 5:**
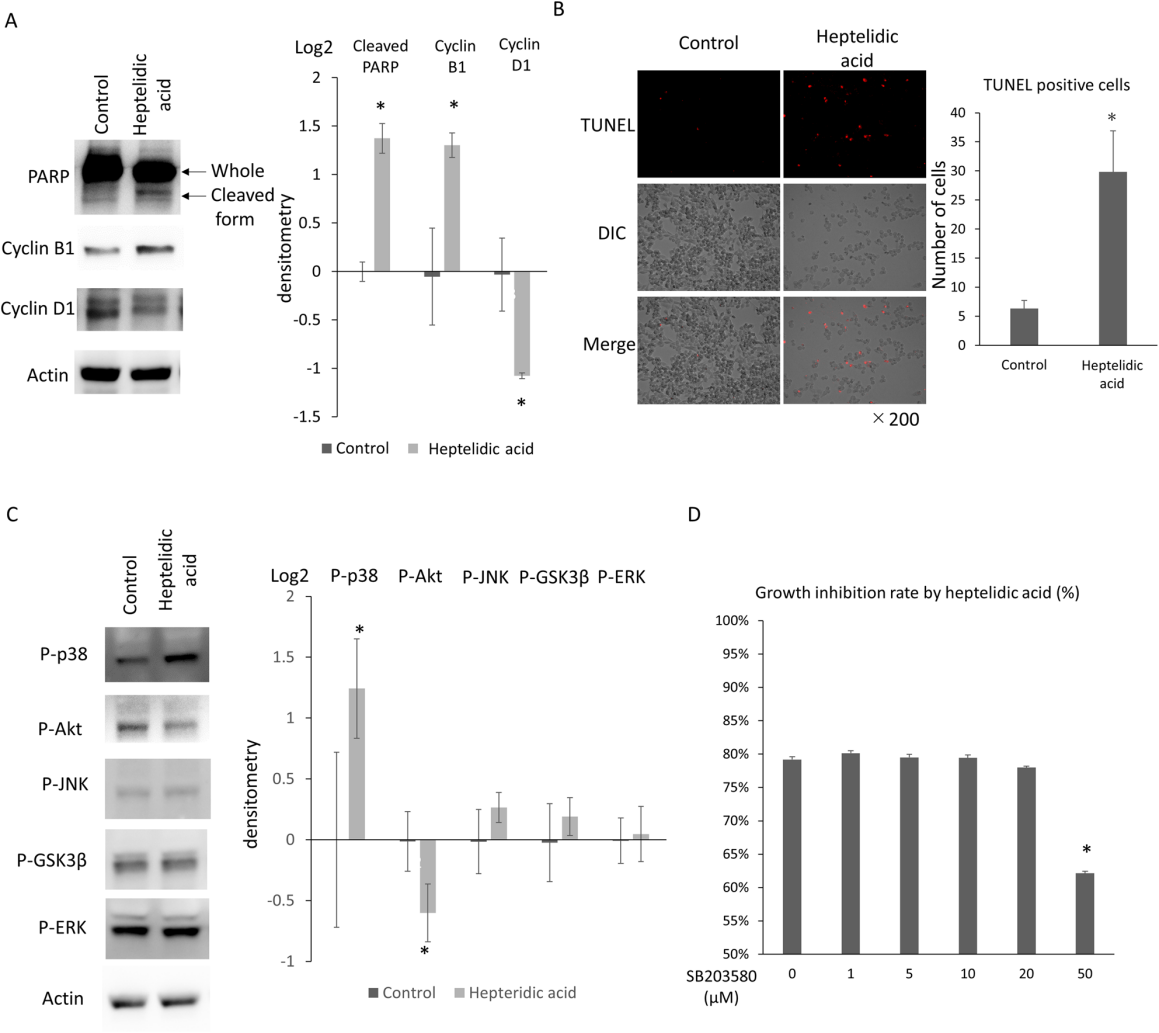
Heptelidic acid exerted anti-tumor effects mediating the p38 MAPK signaling pathway in pancreatic cancer cells. A Western blotting analysis indicated that the expression of cleaved PARP and cyclin B1 was increased, and that of cyclin D1 was decreased in SUIT-2 cells by treatment with 1 μg/mL of heptelidic acid (**A**). TUNEL staining showed that the DNA fragmentation of SUIT-2 cells was induced by treatment with 1 μg/mL of heptelidic acid (**B**). Western blotting showed that the phosphorylation of p38 MAPK was augmented while that of Akt was attenuated by treatment with 1 μg/mL heptelidic acid (**C**). The SRB assay showed that the growth inhibition rate of heptelidic acid was decreased by treatment with SB203580 (**D**). The error bars show the standard deviation (S.D.) (**A**; p-p38; n = 6, p-Akt, p-JNK, p-GSK3β, p-ERK; n = 3) (**C**,**D**; n = 3) (**B**; n = 4).

Western blotting indicated the augmentation of cleaved PARP and cyclin B1 and downregulation of cyclin D1 (Fig. 5A), suggesting that heptelidic acid exerted tumor-suppressive functions by mediating the dysregulation of the cell cycle and induction of apoptosis. Likewise, TUNEL staining revealed that heptelidic acid induced apoptotic reactions, including DNA fragmentation, in pancreatic cancer cells (Fig. 5B). To clarify the intracellular significance of heptelidic acid treatment, the phosphorylation of p38, Akt, JNK GSK3β and ERK was assessed. Western blotting indicated that levels of phosphor-p38 were significantly increased, while levels of phosphor-Akt were significantly decreased (Fig. 5C). To investigate whether or not heptelidic acid induced an tumor-suppressive effect by p38 activation, SB203580, which is an inhibitor of p38 MAPK signaling, was administered to SUIT-2 cells, and the cell growth inhibition effect of heptelidic acid was assessed. An SRB assay indicated that the growth suppression rate by heptelidic acid was significantly decreased by treatment with SB203580 (Fig. 5D), suggesting that heptelidic acid exerted its tumor-suppressive effect via the induction of apoptosis of pancreatic cancer cells by p38 MAPK signaling transduction.

## Discussion

**Figure 6:**
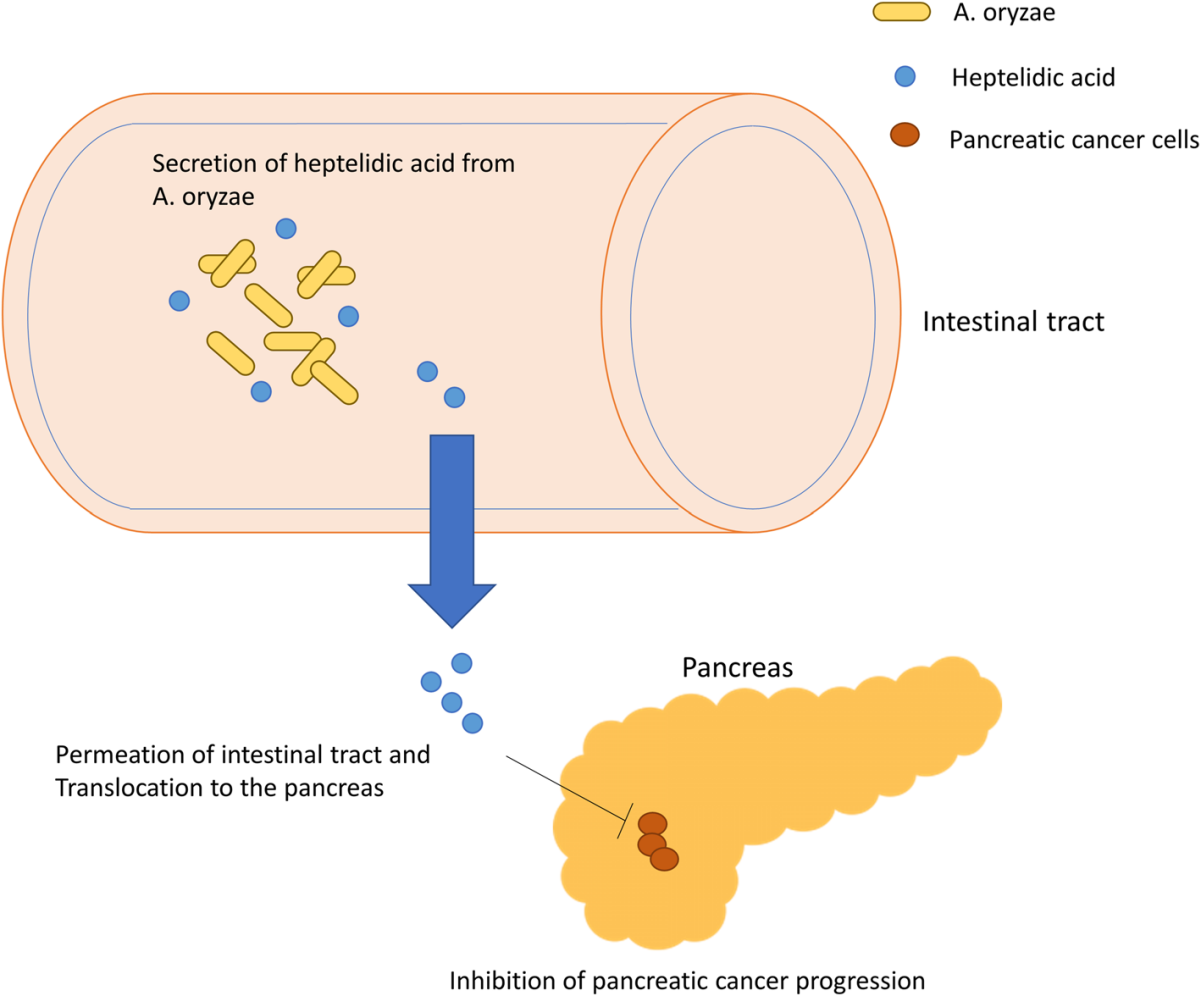
A schematic illustration of the regulatory systems of pancreatic cancer mediating *A. oryzae*. Heptelidic acid secreted by *A. oryzae* can circulate throughout the intestinal tract to enter the host whole body and thereafter it translocates to the pancreas, where it then inhibits the growth of pancreatic cancer.

The present study revealed that conditioned media of *A. oryzae* exerted a strong tumor-suppressive effect against pancreatic cancer cells, suggesting that *A. oryzae* released tumor-suppressive molecules into the conditioned media. After the separation of the conditioned media by several columns, heptelidic acid was enriched in the tumor-suppressive fraction. The tumor-suppressive effects of heptelidic acid were confirmed in pancreatic cancer cells in vitro and in an in vivo xenograft model, and the mechanisms underlying the tumor-suppressive functions were shown to be apoptosis mediation followed by the activation of the p38 MAPK signaling pathway. Through our study, we demonstrated the mechanism underlying the host-microbe communication that brings about health benefits, including anti-tumor effects in organs separate from the gastrointestinal tract, such as the pancreas, through mediation by probiotic-derived molecules (Fig. 6).

*A. oryzae* has been used to produce fermented food, such as soy sauce, and fermented liquor and is known to exert anti-tumor effect for intestinal cancer cells. However, the mechanism underlying this bacterial anti-tumor effect has been unclear. We identified several anti-tumor molecules produced by bacteria, so the effect *A. oryzae* was thought to be mediated by bacterial bioactive molecules. After the separation of *A. oryzae* culture supernatant by HPLC, heptelidic acid was identified in the isolated tumor-suppressive fraction by LC–MS and an NMR analysis. Our xenograft study showed the strong tumor-suppressive effect of heptelidic acid in vivo. TUNEL staining and Western blotting revealed that heptelidic acid induced the apoptosis of pancreatic cancer cells. In addition, the cell cycle-related molecules cyclin D1 and B1 were dysregulated by treatment with heptelidic acid, suggesting that heptelidic acid induced an abnormal cell cycle, resulting in the accumulation of cell cycle-arrested cells and the subsequent apoptosis of pancreatic cancer cells.

Heptelidic acid was first identified as a sesquiterpene antibiotic derived from three strains of fungi: *Gliocladium virens, Chaetomium globosum and Trichoderma viride*^26^. It was also isolated from the bacterial culture of *T. koningii* as a glyceraldehyde 3-phosphate dehydrogenase (GAPDH) inhibitor, suppressing ATP generation in mouse carcinoma FM3A cells^27^. Several groups have shown that *A. oryzae* produce heptelidic acid and biogenesis-related gene clusters^24,28,29^. Recently, Liberti et al. revealed that heptelidic acid exerted anti-tumor effects in cells that depended on the Warburg effect (WE)^30^. Likewise, p38 MAPK signaling is known to be a key regulatory signaling pathway of glycolysis^31–33^ in pancreatic cancer cells and our findings showed that heptelidic acid exerted an anti-tumor effect which was mediated by p38 signal transduction. These findings suggest that anticancer therapy, especially pancreatic cancer treatment, utilizing heptelidic acid may prove effective.

Our SRB assay showed the anti-tumor effect when heptelidic acid was directly administered to pancreatic cancer cells. Of note, to exert its anti-tumor effect in distant organs, such as pancreatic cancer, from the intestinal tract, heptelidic acid must transit the intestinal tract and reach the pancreatic tissue. A previous investigation showed that microbial metabolites, such as short-chain fatty acids (SCFAs), but not the bacterial body, were able to pass through the intracellular space of the intestinal tract and modulate the cellular functions of distant organs, including the pancreas^34^. An MS analysis showed that the molecular size of heptelidic acid was only 280.13. Furthermore, heptelidic acid was considered to have high lipophilicity because the heptelidic acid was enriched when separated by a reverse-phase column, which was selected based on the degree of lipophilicity. These suggests that heptelidic acid can pass through the cell membrane, escape the intestinal tract, enter the blood stream and reach cancer cells in extra-intestinal organs, including the pancreas.

Western blotting showed that the cell cycle regulator cyclin B1 was significantly augmented by heptelidic acid treatment. The expression of cyclin B1 is known to increase in the G2 phase, where it forms a complex with Cdk1. The formation of Cyclin 1-CDK1 complex is an indispensable step for the cell cycle to transform into the M phase^35,36^. Heptelidic acid also irreversibly inhibits GAPDH^27^, which promotes the production of ATP mediating glycolysis, thereby accelerating the cell cycle by modulating the cyclin B1-cdk1 activity^37^. These findings indicate that cell cycle arrest, especially in the G2/M phase, was induced by heptelidic acid, resulting in an increase in cyclin B1.

It was previously reported that metabolic syndrome, including diabetes, is associated with the development of pancreatic cancer^16^. Interestingly, microbiota transplantation from obese mice to germ-free mice reportedly resulted in a significant increase in total body fat^38^, and fecal transplantation from pancreatic cancer patients with a short-term survival (STS) and long-term survival differentially influenced the immune response and natural history of the disease^21^. Likewise, soybean foodstuffs fermented by *A. oryzae* exhibited a preventative effect on obesity by modulating the insulinotropic action in diabetic rats^39^. These findings suggest that the probiotic *A. oryzae* produces anti-obese molecules as well as anti-tumor molecules, such as heptelidic acid, helping prevent diabetic diseases and inhibit cancer growth.

In conclusion, we showed that heptelidic acid released by *A. oryzae* exerted anti-tumor functions by inducing apoptosis mediated by p38 signaling activation in pancreatic cancer cells. Furthermore, heptelidic acid exerted an anti-tumor effect, showing that intestinal bacteria, including *A. oryzae,* can inhibit cancer cell growth in extra-intestinal organs via the transfer of bacteria-derived anti-tumor molecules, which is considered a novel anti-tumor mechanism of commensal bacteria.

## Supplementary Information

Supplementary Information.
