# Supplementary material for: Probiotic Aspergillus oryzae produces anti-tumor mediator and exerts anti-tumor effects in pancreatic cancer through the p38 MAPK signaling pathway
Source: Sci Rep. 2021 May 26;11:11070. doi: 10.1038/s41598-021-90707-4 (PMC8154913; doi:10.1038/s41598-021-90707-4)
Supplement: Supplementary file 1 — Supplementary Information. [file 41598_2021_90707_MOESM1_ESM.pdf]

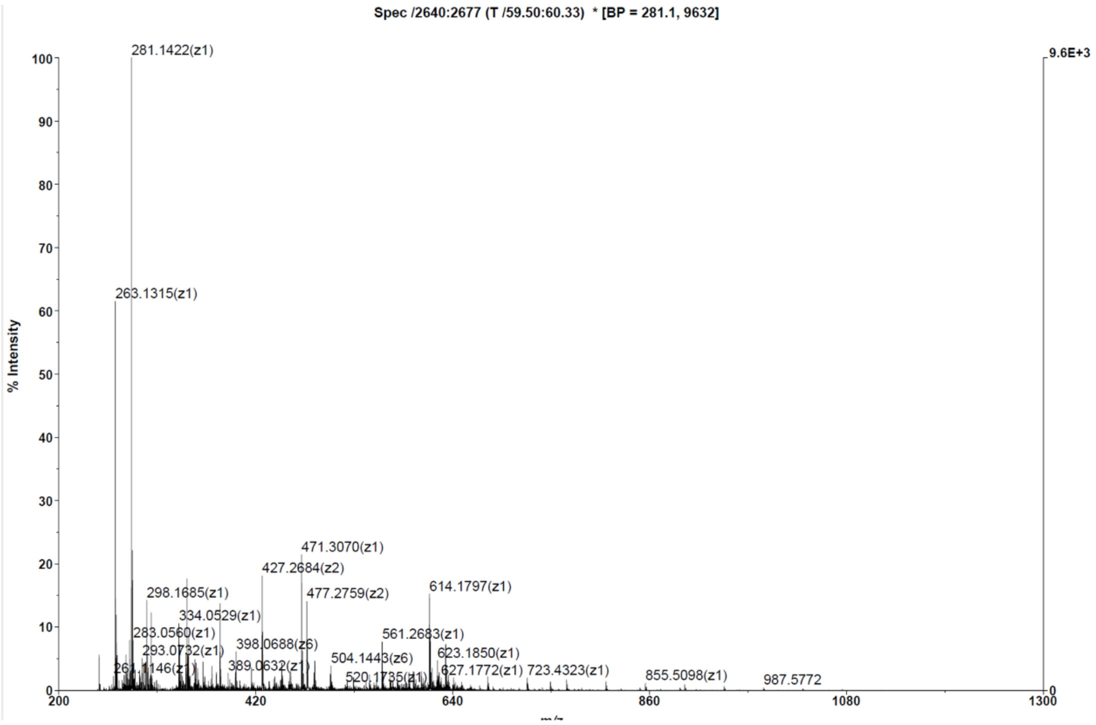

Supplementary Figure 1. The spectrum of isolated fraction by LC-MS analysis

An LC-MS analysis showed the mass of the molecule contained in the final fraction.

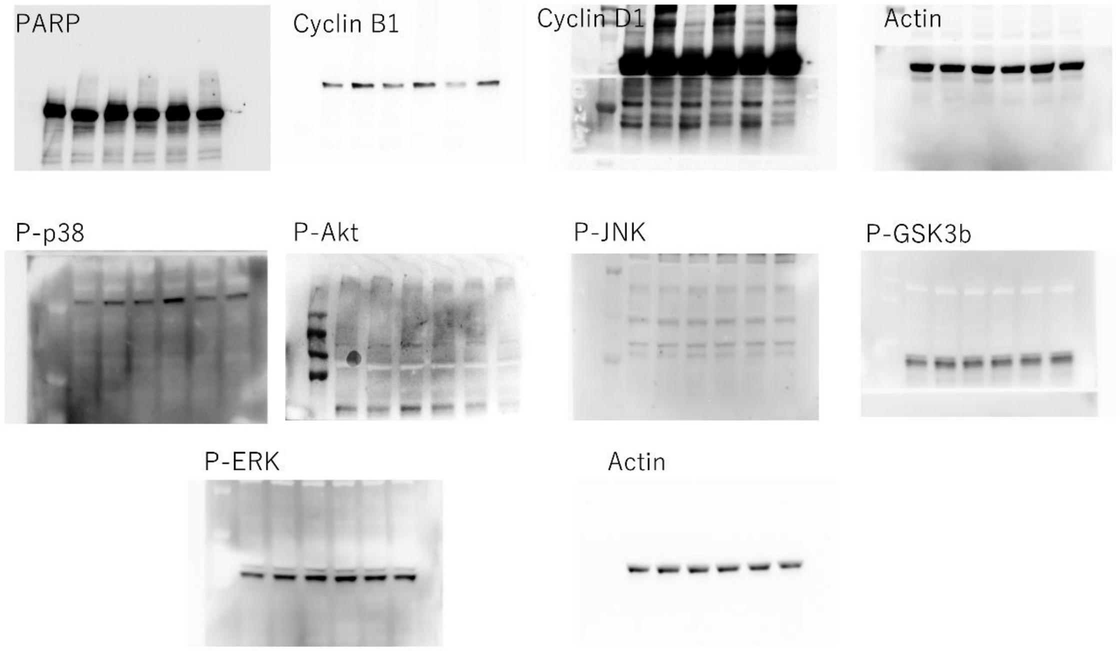

Supplementary Figure 2. Unprocessed images of western blots
